# Robot-assisted microsurgical vasovasostomy: the learning curve for a pure microsurgeon

**DOI:** 10.1007/s11701-018-0888-0

**Published:** 2018-10-31

**Authors:** Parviz K. Kavoussi, Charlie Harlan, Keikhosrow M. Kavoussi, Shahryar K. Kavoussi

**Affiliations:** 1 Austin Fertility and Reproductive Medicine/Westlake IVF, 300 Beardsley Lane, Building B, Suite 200, Austin, TX 78746 USA; 2 St. David’s South Austin Medical Center, 901 West Ben White Blvd, Austin, TX 78704 USA

**Keywords:** Vasectomy reversal, Vasovasostomy, Robotic, Microsurgery

## Abstract

Vasovasostomy success rates improved with the application of the operative microscope in 1975. The robotic platform offers potential advantages including: a stable, ergonomic, scalable control system with three-dimensional visualization and magnification, the elimination of physiological tremor, and simultaneous control of three instruments and a camera. A previous publication revealed a fellowship-trained microsurgeon (PKK) could transition to robot-assisted microsurgical vasovasostomy (RAVV) with comparable outcomes. The objective of this current study was to evaluate the learning curve for the purely trained microsurgeon transitioning to RAVV. A retrospective chart review was performed of a microsurgeon’s first 100 RAVVs evaluating the learning curve for patency rates, anastomosis times, operative times, and sperm concentrations at the initial postoperative semen analyses. Cases were stratified into four groups by 25 case intervals. There were no statistically significant differences in patency rates or postoperative sperm concentrations between the groups over time. There were differences in anastomosis times between groups 1 and 2, as well as between groups 2 and 3, and there were differences in operative times between groups 2 and 3. High-percentage patency rates are achievable very early in the transition from pure microsurgical vasovasostomy to RAVV across wide ranges of obstructive intervals. Postoperative mean sperm concentrations in the initial semen analyses after RAVV are consistent over time. For a single microsurgeon not formally trained in robotic microsurgery, 75 RAVV cases were required to optimize and plateau in anastomosis times and 75 cases were required to optimize operative times.

## Introduction

Approximately 500,000 men undergo vasectomy for contraception annually in the United States. Of those men, an estimated 6% will seek vasectomy reversal (VR) to re-establish fertility potential [1, 2]. Although the ultimate goal of VR is live birth, technical success with VR has been defined as patency, and patency rates have been associated with obstructed interval since the vasectomy, intraoperative findings of the quality of the vasal fluid examined from the testicular end of the vas deferens, as well as the presence of sperm or sperm fragments in the vasal fluid intraoperatively, surgical technique, and the training and experience of the surgeon [3–7]. Anastomosis times and operative times play an important practical role in cost effectiveness, throughput of surgical cases in a day, surgeon fatigue, and patient time under anesthesia for men undergoing VR. Patency, or success, is defined by the findings in the postoperative semen analysis.

VR technology has evolved over time. Initially VR was performed with the naked eye, VR then progressed to the use of optical loupe lenses which offered some level of magnification. Significant improvements in outcomes were fostered with the advent of the operative microscope with its use for microsurgical VR in 1975 [8]. Since that time, there have been some minor changes in instrumentation used for VR, but little change has been made in the way of alternate magnification sources or significantly different technology [9]. That is until relatively recently with the application of the robotic platform for use with microsurgery and specifically with robot-assisted vasectomy reversal (RAVR). With the utilization of a new technology applied to an established operation, the question of outcomes must be answered, which has been evaluated with animal and clinical human studies [10–14], and it is of importance that the learning curve for the application of such technology to an existing procedure is evaluated, which was the aim of this study.

## Materials and methods

After Institutional Review Board exemption was obtained, a retrospective chart review was performed on the first 100 robot-assisted microsurgical vasovasostomies (RAVV) by a single fellowship-trained microsurgeon (PKK). All patients underwent informed consent with standard operative consents for vasectomy reversal. Data on robot-assisted vasectomy reversal outcomes from prior published studies, as well as the operating surgeon’s patency rates maintained for quality assurance for RAVR, were shared with consenting patients as the series of cases progressed. The surgical technique to perform RAVV has been previously described [15]. The cases were divided into four categories, chronologically with 25 consecutive RAVV cases in each timeframe category. The data was limited to RAVV; therefore, robot-assisted microsurgical vasoepididymostomies were excluded for uniformity. Redo surgeries such as failures after previous VR attempts from other surgeons were excluded for uniformity. Factors including obstructive intervals since vasectomy, initial patency rates, anastomosis times, operative times, and sperm concentrations in postoperative semen analyses were assessed and compared for each consecutive time frame category. Operative times were defined as the time from incision to completion of closure. Initial patency was defined as sperm concentration equal to or greater than 1 million sperm per mL of semen at 6 weeks post VR. Patients with sperm concentrations of zero at 6 weeks were considered early failure, although they were followed out for the following 6 months when the patients maintained follow-up visits. Sperm concentrations were compared at this same time interval for uniformity as some patients were lost to follow-up beyond the 6 week post RAVV semen analysis.

Statistical analyses were performed with a one-way ANOVA and Student’s t tests was performed on the categories revealing a statistically significant difference by the ANOVA. A *p* value of < 0.05 was considered statistically significant.

## Results

The first 100 RAVV cases were divided into four categories, each containing 25 consecutive RAVV cases. Group 1 included RAVV cases 1 through 25, group 2 included cases 26 through 50, group 3 included cases 51 through 75, and group 4 included cases 76 through 100. There was no significant difference between the obstructed intervals between the groups (*p* = 0.18), no significant difference in initial patency rates between the groups (*p* = 0.93), and no significant difference in initial sperm concentrations between the groups (*p* = 0.20). There were statistically significant differences by ANOVA in anastomosis times (*p* = 0.00001) and operative times (*p* = 0.02) (Table 1). There were statistically significant differences in anastomosis times between group 1 and group 2 (*p* = 0.004) and between group 2 and group 3 (*p* = 0.036). Anastomosis times did not reveal a significant difference between group 3 and 4 (*p* = 0.804). The only case volume category that revealed a statistically significant difference in operative times was between group 2 and group 3 (*p* = 0.036) (Table 2).

**Table 1:** ANOVA results for obstructive intervals since vasectomy in years, patency rates, anastomosis times in minutes, operative times in minutes, and sperm concentrations post RAVV in millions of sperm per mL of semen

	Group 1 (*n* = 25)	Group 2 (*n* = 25)	Group 3 (*n* = 25)	Group 4 (*n* = 25)	*p* value
Obstructive interval mean (SD)	8.9 (5.9)	9.1 (6.3)	7.6 (3.7)	10.9 (4.8)	0.18
Patency rates mean (SD)	92% (0.3)	92% (0.3)	92% (0.3)	96% (0.2)	0.93
Anastomosis time mean (SD)	74.6 (13.8)	63.4 (11.9)	55.9 (11.9)	56.6 (7.4)	< 0.00001
Operative time mean (SD)	121.9 (15.0)	121.2 (21.0)	107.8 (23.0)	105.0 (22.0)	0.02
Sperm concentration mean (SD)	26.2 (37.3)	28.4 (33.9)	21.9 (26.7)	25.7 (18.7)	0.89

**Table 2:** Anastomosis and operative times between consecutive RAVV groups

Group 1 mean (SD)	Group 2 mean (SD)	*p* value
Anastomosis times between groups 1 and 2 in minutes		
74.6 (13.8)	63.4 (11.9)	0.004

Group 2 mean (SD)	Group 3 mean (SD)	*p* value
Anastomosis times between groups 2 and 3 in minutes		
63.4 (11.9)	55.9 (11.9)	0.036

Group 3 mean (SD)	Group 4 mean (SD)	*p* value
Anastomosis times between groups 3 and 4 in minutes		
55.9 (11.9)	56.6 (7.4)	0.804

Group 1 mean (SD)	Group 2 mean (SD)	*p* value
Operative times between groups 1 and 2 in minutes		
121.9 (15.0)	121.2 (21.0)	0.892

Group 2 mean (SD)	Group 3 mean (SD)	*p* value
Operative times between groups 2 and 3 in minutes		
121.2 (21.0)	107.8 (23.0)	0.036

Group 3 mean (SD)	Group 4 mean (SD)	*p* value
Operative times between groups 3 and 4 in minutes		
107.8 (23.0)	105.0 (22.0)	0.662

## Discussion

Since the application of the operative microscope for use with microsurgical vasovasostomies in 1975, there has been little advancement in the technology to help advance the surgeons ability to perform this technically demanding operation [8]. The robotic platforms utilization for microsurgical vasovasostomy is the first major advancement in tools available to microsurgeons to perform microsurgical vasovasostomies. The single microsurgeon performing the RAVVs from which this data was extracted, was formally trained in microsurgical vasectomy reversal in a two-year reproductive urology fellowship program following urology residency training with very minimal robotic console training time, as this was the timeframe when robotic surgery was just coming to the forefront for laparoscopy-assisted robotic prostatectomy. It has been established that the fellowship-trained microsurgeon can transition to RAVR with comparable outcomes [15]. The next measure to consider is not only outcomes but the learning curve for a purely microsurgery-trained reproductive urologist with minimal robot training, which was assessed in this study.

Patency rates were assessed and were equivalent at 92% for the first three time interval groups, and the patency rate increased to 96% after the 75th case. Although this may arguably be a clinically significant difference, it is not a statistically significant improvement in patency after the 75th case. As obstructive intervals did not reveal differences between the groups, each group included men with very short to very long obstructive intervals from the time of vasectomy, therefore, these patency rates are not stratified by obstructive intervals, as is the manner which patency rates are commonly reported.

The same surgeon’s anastomosis times and operative times with pure microsurgical VRs were published in a previous study at 64 min and 141 min, respectively [15]. This is in comparison to the current studies RAVV anastomosis times for the four consecutive groups of 25 cases each, being 74.6 min, 63.4 min, 55.9 min, and 56.6 min, respectively; and RAVV operative times for the four groups being 121.9 min, 121.2 min, 107.8 min, and 105.0 min, respectively. This comparison reveals that a fellowship-trained microsurgeon achieves faster RAVV anastomosis times than microsurgical VR times after 25 RAVV cases, and faster RAVV operative times than microsurgical operative times even in the first 25 cases.

Although this data suggests that formally trained microsurgeons can transition to RAVV with a relatively quick learning curve, there should be caution in interpreting this as an appropriate procedure for robotic surgeons without microsurgical training. The minimal touch microsurgical tissue handling techniques and skills acquired with microsurgical training are not obviated by the robotic platform, as such skills are applied to the robotic platform.

Limitations to this study include the retrospective nature with inherent biases as well as this learning curve being based on the progression of a single surgeon, where there may be variability depending on the individual surgeon’s skill and pace of learning with a new technology. Another limitation is the data is only extended over the first 100 cases of RAVV performed by this single surgeon; however, the surgeon maintains quality assurance data and did not see further progression beyond the 100th case in the categories assessed, so the anastomosis and operative times did plateau following the case volumes that demonstrated statistically significant improvements in these metrics as presented in this study.

## Conclusions

High-percentage patency rates are achievable very early in the transition from pure microsurgical vasovasostomy to RAVV. Postoperative mean sperm concentrations in the initial semen analyses after RAVV are consistent over time. For a single microsurgeon not formally trained in robotic surgery, 75 RAVV cases were required to optimize and plateau in anastomosis times and 75 cases were required to optimize operative times.
